# Circulating Levels of VEGF, mTOR and MAPK in Patients With Port‐Wine Stains

**DOI:** 10.1111/jocd.70232

**Published:** 2025-05-29

**Authors:** Yuanbo Huang, Panyu Wu, Jun Yang, Mingye Bi, Lei Cao, Lei Wang

**Affiliations:** ^1^ Department of Dermatology Affiliated Women's Hospital of Jiangnan University Wuxi China; ^2^ Department of Dermatology The Affiliated Wuxi People's Hospital of Nanjing Medical University Wuxi China; ^3^ Department of Dermatology, Wuxi No. 2 People's Hospital Jiangnan University Medical Center Wuxi China

**Keywords:** ELISA, MAPK, mTOR, port‐wine stains, VEGF

## Abstract

**Background:**

Vascular endothelial growth factor (VEGF), mammalian target of rapamycin (mTOR) and mitogen‐activated protein kinase (MAPK) pathways play important roles in the pathogenesis of port‐wine stains (PWS). However, reports on circulating levels of VEGF, mTOR, and MAPK in PWS patients are rare.

**Objective:**

To investigate the circulating levels of VEGF, mTOR, and MAPK in PWS patients compared to healthy controls.

**Methods:**

Thirty patients with PWS (15 with hypertrophic PWS and 15 with flat PWS) and 15 healthy controls were included. Human VEGF, mTOR, and MAPK enzyme‐linked immunosorbent assay (ELISA) kits were used to test plasma samples, and concentration values were calculated. Differences in the concentrations of VEGF, mTOR, or MAPK between the two groups were analyzed using *t*‐tests or analysis of variance.

**Results:**

The circulating levels of VEGF, mTOR, and MAPK in the PWS group were significantly higher than in the healthy control group, with concentration values of 1159.76 ± 162.83 pg/mL vs. 762.18 ± 199.88 pg/mL, 219.26 ± 54.25 ng/L vs. 108.93 ± 52.47 ng/L, 11.41 ± 2.53 ng/L vs. 6.35 ± 2.42 ng/L (*p* < 0.0001, *p* < 0.0001 and *p* < 0.0001; respectively). VEGF circulating levels in the hypertrophic PWS group were significantly higher than in the flat PWS group (1217.97 ± 141.17 pg/mL vs. 1101.55 ± 166.52 pg/mL, *p* = 0.04). However, the circulating levels of mTOR or MAPK between the hypertrophic PWS and flat PWS groups were no significant differences (*p* = 0.87, *p* = 0.42; respectively).

**Conclusion:**

The circulating levels of VEGF, mTOR, and MAPK were significantly higher in PWS patients than healthy controls, with VEGF levels being higher in hypertrophic PWS than in flat PWS.

## 1 Introduction

Port‐wine stains (PWS) are common capillary malformations with a prevalence of 3%–8% [1, 2]. These lesions often appear on the exposed areas, affecting the appearance, psychological well‐being, and even function. The exact pathogenesis of this disorder remains unclear, but possible mechanisms include somatic gene mutations [3, 4], a decrease in perivascular nerve elements [5, 6], the coexistence of Eph receptor B1 and ephrin B2 [7], and a deficiency of SMA expression in pericytes [8]. Additionally, vascular endothelial cell growth factor (VEGF), mammalian target of rapamycin (mTOR), and mitogen‐activated protein kinase (MAPK) signaling pathways play important roles in the pathogenesis and progression of PWS [9, 10]. Most studies focused on PWS lesions, but obtaining tissue samples is invasive and requires approval from both patients and ethics committees.

PWS usually progresses to hypertrophic or nodular form after the age of 30 [11]. However, some patients experience subcutaneous tissue lesions, or even bone hypertrophy, before school age [12]. We hypothesized that factors related to proliferation in the blood, such as VEGF, mTOR, and MAPK, may be abnormal. Previous research has detected elevated mTOR expression in the blood of patients with hyperlipidemia using enzyme‐linked immunosorbent assay (ELISA) analysis [13]. To investigate the circulating levels of VEGF, mTOR, and MAPK in PWS patients and determine whether they are associated with the pathogenesis and progression of PWS, we conducted this study.

## 2 Methods

### 2.1 Participants

PWS patients were selected from the dermatology department, while healthy controls were chosen from the Health Examination Centre of the same hospital. Inclusion criteria were: ① PWS diagnosed by two dermatologists; ② Total PWS lesion size greater than 40 cm^2^; ③ No treatment for PWS within the last six months. Exclusion criteria were: ① Presence of chronic systemic diseases, such as autoimmune diseases, infectious diseases, cardiovascular diseases, metabolic diseases, tumors; ② Acute infection within the last month, such as respiratory tract infection. The control group had no history of chronic disease or acute infection within the last month, and their blood cell count, routine urinalysis, liver function tests, renal function tests, blood lipid levels, blood glucose level, and electrocardiogram results were normal. Written informed consent was obtained from all subjects and their guardians. This research was approved by the ethics committee of the hospital (KS202056).

### 2.2 Sample Collection

Venous blood was collected from each subject. The samples were centrifuged (10 min, 3500 rpm) within two hours of collection. The plasma was then removed and stored at −80°C.

### 2.3 ELISA Analysis

Plasma levels of VEGF, mTOR, and MAPK were measured using ELISA kits (MSK biological technology co., LTD, Wuhan, China). All samples were assayed in duplicate and read at 450 nm.

### 2.4 Statistical Analysis

Blood levels in each group are expressed as mean ± standard deviation. T‐test or analysis of variance was used to calculate the differences between two groups. *p‐*values < 0.05 were considered statistically significant. All analyses were conducted using SPSS 20.

## 3 Results

### 3.1 General Information of PWS Patients and Healthy Controls

A total of 30 patients with PWS (15 with hypertrophic PWS and 15 with flat PWS) and 15 healthy controls were included. The average age of the PWS group and the control group was 24.77 ± 9.11 and 18.53 ± 4.10 years, respectively. General information of all participants is presented in Table 1.

**TABLE 1:** General information and plasma levels of VEGF, mTOR, and MAPK in all participants.

Category	Hypertrophic PWS	Flat PWS	Healthy control
*N*	15	15	15
Gender (male/female)	6/9	2/13	8/7
Age (year)	23.8 ± 12.18	33.0 ± 25.8	18.53 ± 4.10
VEGF (pg/mL)	1217.96 ± 141.17	1101.55 ± 166.52	762.18 ± 199.88
mTOR (ng/L)	217.61 ± 53.16	220.90 ± 57.14	108.93 ± 52.47
MAPK (ng/mL)	11.80 ± 2.55	11.03 ± 2.55	6.35 ± 2.42

### 3.2 Plasma Levels of VEGF in PWS Patients and Healthy Controls

**FIGURE 1:**
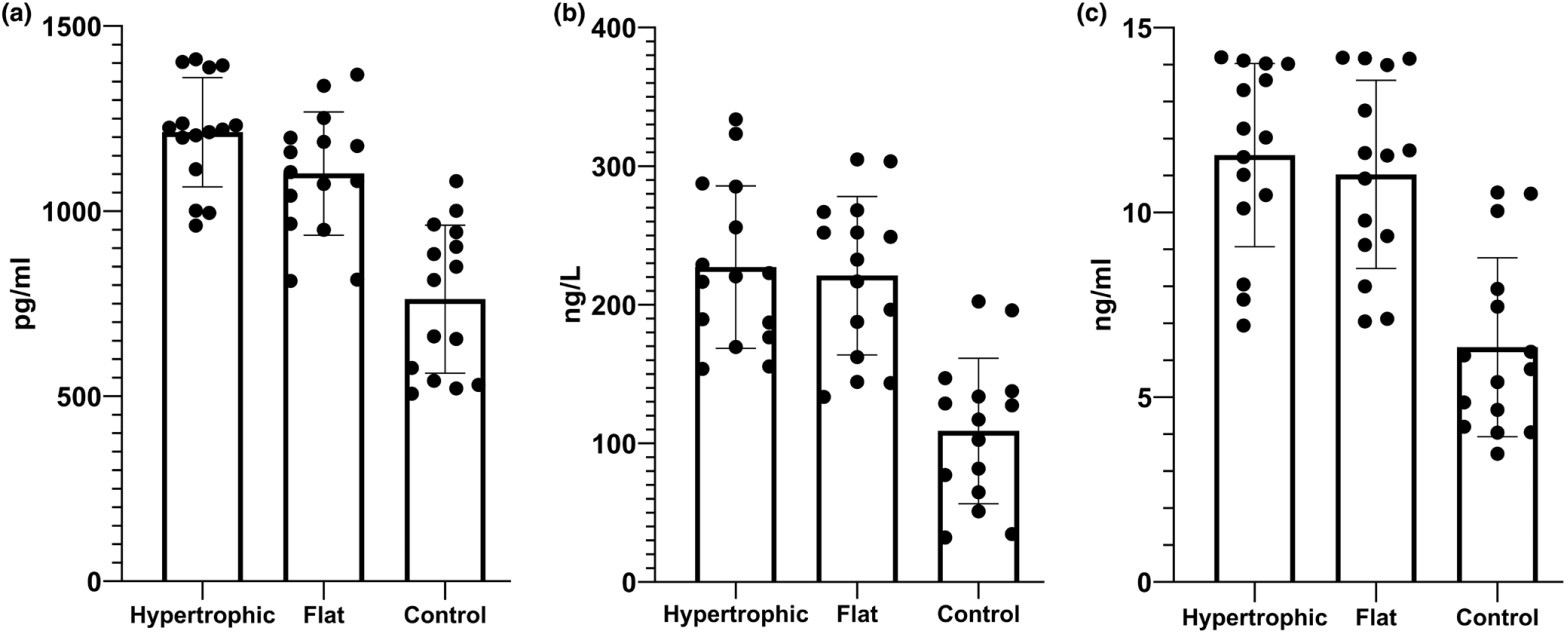
(a) Circulating levels of VEGF in PWS patients and healthy controls (1217.97 ± 141.17 vs. 1101.55 ± 166.52 vs. 762.18 ± 199.88), (b) circulating levels of mTOR in PWS patients and healthy controls (217.61 ± 53.16 vs. 220.90 ± 57.14 vs. 108.93 ± 52.47), (c) circulating levels of MAPK in PWS patients and healthy controls (11.80 ± 2.55 vs. 11.03 ± 2.55 vs. 6.35 ± 2.42).

The plasma levels of VEGF in the PWS group were 1159.76 ± 162.83 pg/mL, compared to 762.18 ± 199.88 pg/mL in the control group, showing a significant difference (*F* = 51.17, *p* < 0.0001). Among PWS patients, the plasma levels of VEGF were 1217.97 ± 141.17 pg/mL in those with hypertrophic PWS and 1101.55 ± 166.52 pg/mL in those with flat PWS, also showing a significant difference (*t* = 2.12, *p* = 0.04). Additionally, VEGF levels in flat PWS patients were obviously higher than healthy controls (*F* = 25.53, *p* < 0.0001). The plasma levels of VEGF in the three groups are shown in Figure 1a.

### 3.3 Plasma Levels of mTOR in PWS Patients and Healthy Controls

The plasma levels of mTOR in PWS patients were 219.26 ± 54.25 ng/L, compared to 108.93 ± 52.47 ng/L in healthy controls, showing a significant difference (*t* = 6.50, *p* < 0.0001). In the hypertrophic PWS group, the plasma levels of mTOR were 217.61 ± 53.16 ng/L, and in flat PWS patients, they were 220.90 ± 57.14 ng/L, with no statistical difference between them (*t* = 0.16, *p* = 0.87). However, there was a statistical difference in mTOR levels between flat PWS patients and healthy controls (F = 31.25, *p* < 0.0001). The plasma levels of mTOR in the three groups are shown in Figure 1b.

### 3.4 Plasma Levels of MAPK in PWS Patients and Healthy Controls

The plasma levels of MAPK in PWS patients were 11.41 ± 2.53 ng/L, compared to 6.35 ± 2.242 ng/L in healthy controls, showing a significant difference (*F* = 41.11, *p* < 0.0001). Among the PWS group, the plasma levels of MAPK were 11.80 ± 2.55 ng/L in those with hypertrophic PWS and 11.03 ± 2.55 ng/L in those with flat PWS; there was no statistical difference (*F* = 0.68, *p* = 0.42). However, MAPK levels in flat PWS patients were also obviously higher than healthy controls (F = 26.59, *p* < 0.0001). The plasma levels of MAPK in the three groups are shown in Figure 1c.

## 4 Discussion

VEGF is the most well‐known proangiogenic factor, discovered by Senger et al. [14]. It includes six isoforms: VEGF‐A to VEGF‐F. VEGF‐A is the main factor promoting tumor vascular growth and is expressed in multiple normal organs, such as lung, kidney, heart, and ovaries. VEGF binds with corresponding receptors and activates p38 MAPK/mTOR, PI3K/AKT/mTOR, or classical MAPK signaling pathways. This binding affects cell proliferation, migration, and vascular permeability, thus promoting angiogenesis and vascular dilation [15]. Studies have revealed that VEGF expression in lesions is associated with the onset and hypertrophy in PWS. Using immunofluorescence staining, researchers revealed that VEGF and VEGF‐R2 are overexpressed in PWS lesions compared to normal skin (*p* < 0.005); however, the type of PWS was not mentioned [16]. Another study showed that the positive expression rates of VEGF in normal skin tissue, flat PWS, hypertrophic PWS, nodular PWS, and limb PWS were 0%, 35.7%, 4.8%, 75%, and 40%, respectively [17]. The positive expression rates of VEGF in normal skin tissue and PWS were significantly different (*p* < 0.05) [17]. The results suggest that VEGF expression is upregulated to varying degrees in different types of PWS, especially in local nodular PWS, which may be related to PWS occurrence and development [17].

Some studies have detected VEGF blood concentrations in patients with PWS (vascular malformation). Zhang et al. found that the VEGF blood levels in proliferating hemangiomas were significantly higher than in involuting hemangiomas, vascular malformations, and normal controls [18]. However, there was no significant difference in VEGF blood levels among patients with involuting hemangiomas, vascular malformations, and healthy controls [18]. Li et al. showed no significant difference in VEGF blood levels between patients with involuting hemangioma and normal controls [19]. El‐Raggal et al. found that VEGF blood levels in proliferating hemangioma, involuting hemangioma, and vascular malformation were significantly higher than normal controls (*p* < 0.001) [20]. These studies suggest that VEGF blood levels in PWS (vascular malformation) are inconsistent across different studies. However, these studies did not mention whether hypertrophic PWS were included, and further research is needed to clarify VEGF blood levels in hypertrophic PWS.

In our study, we detected VEGF blood levels in flat PWS, hypertrophic PWS, and healthy controls. We found that these levels are significantly higher in PWS patients compared to healthy controls. Stratification analysis showed that VEGF blood levels in flat PWS were significantly higher than in healthy controls, and these levels in hypertrophic PWS were significantly higher than in flat PWS. This indicates that VEGF may be associated with both the onset and progression of PWS lesions. Detecting VEGF levels in blood may help predict the hyperplasia of PWS and provide clues for developing new therapies, such as targeted therapy based on VEGF inhibitors. However, the underlying mechanism of VEGF involvement in the pathogenesis and development of PWS needs further investigation.

mTOR is an atypical serine/threonine protein kinase that was identified following the discovery of rapamycin, isolated from soil bacteria on Rapanui Island in 1970 [21]. mTOR can produce two different functional complexes: mTOR C1 and mTOR C2. mTOR C1 is crucial for cell growth and metabolism, while mTOR C2 regulates proliferation and survival [22, 23]. mTOR is involved in multiple signaling pathways, including the PI3K/AKT/mTOR pathway, which plays an essential role in tumor formation and development, and the LKB1‐AMPK‐TSC‐mTOR pathway, which regulates energy metabolism. Several studies have revealed that mTOR plays an important role in the progression of PWS, mainly through the PI3K/AKT/mTOR pathway, which can be activated by overexpression of the VEGF receptor or TIE gene mutations [15].

Researchers found that the p70S6 and eIF4EBP1 in the mTOR signaling pathways gradually increased from normal skin to flat type PWS, hypertrophic PWS, and nodular PWS; and positively correlated with the progress of PWS [24]. Western blotting showed that the expression of p70S6 and eif4EBP1 in this disease was higher than that in healthy skin. Furthermore, these expressions were higher in hypertrophic and nodular PWS than in flat PWS [24]. The activation of the mTOR signaling pathway was located in the endothelial cells of the malformed vessels of PWS [24]. Scholars suggested that the mTOR signaling pathway is gradually activated during the development of PWS, and this activation may be related to the thickening and nodule formation in PWS [24]. Rapamycin, an mTOR inhibitor, has shown some efficacy in treating PWS, but its effectiveness remains controversial [25, 26, 27]. Currently, the blood levels of mTOR in PWS patients have not been reported. In our study, mTOR levels in the blood of PWS patients (including hypertrophic PWS and flat PWS) were significantly higher than that in the control group, suggesting that increased mTOR levels may be associated with the onset of PWS. However, there was no significant difference in mTOR levels between hypertrophic and flat PWS, indicating that mTOR levels may not be associated with the thickening progression of PWS.

The MAPK signal pathway is a crucial pathway in the eukaryotic signaling network, controlling several basic cellular processes: proliferation, differentiation, migration, apoptosis, and stress response [28]. This pathway also plays an important role in endothelial cell function, with gene deficiency in the pathway associated with vascular defects during embryogenesis [29]. Studies have shown that the coexpression of EphB 1/EfnB 2 and the overexpression of VEGF‐A and VEGFR2 can activate MAPK [15]. The Ras, Raf, MEK, and ERK proteins are key factors in this pathway. Knockout research indicated that the MEK5/ERK5 MAPK cascade is essential for the embryonic development of blood vessels and the integrity of mature blood vessels [30, 31]. Dysregulation of MAPK signaling plays a role in the pathogenesis and progression of PWS/SWS [15]. However, the correlation between blood levels of MAPK and PWS has not been reported. In our study, we found that MAPK blood levels in PWS patients were increased, indicating an association with the onset of PWS. However, there was no significant difference in MAPK levels between hypertrophic and flat PWS, suggesting that the MAPK blood levels may not be associated with the thickening progression of PWS.

Previous studies have confirmed the function of these signaling pathways. VEGF exerts its biological effects by binding to VEGF receptors on target cells, and the overexpression of either VEGF or VEGFR activates downstream signal transduction pathways, including the PI3K/AKT/mTOR, p38‐MAPK/mTOR, and PKC/MEK/ERK (classic MAPK) pathways. The PI3K/AKT/mTOR pathway can also stimulate VEGF secretion through both hypoxia‐inducible factor‐1‐dependent and independent mechanisms. The relationship between these pathways is complex. Both the PI3K/AKT/mTOR and MAPK signaling pathways play crucial roles in vascular development. It is widely accepted that the PI3K/AKT/mTOR signaling pathway can induce both angiogenesis and vascular permeability, while the MAPK signaling pathway primarily induces angiogenesis [32]. These pathways promote endothelial cell proliferation, migration, survival, permeability, differentiation, and modulation of cell‐adhesion molecules, thereby facilitating angiogenesis and the dilation of blood vessels [33]. In this study, the further elevation of blood VEGF levels was associated with lesion thickening, suggesting that this protein plays a key role in the progression of PWS.

## 5 Conclusion

In this study, the circulating levels of VEGF, mTOR, and MAPK were significantly higher in PWS patients compared to healthy controls, with VEGF levels being higher in hypertrophic PWS than in flat PWS. This implies that the increased circulating levels of VEGF, mTOR, and MAPK may be associated with the pathogenesis of PWS, and the further increased expression of VEGF may be related to the thickening progression of PWS. However, this study is preliminary, and further verification is needed to reveal the underlying mechanisms, which may provide a theoretical basis for the treatment of PWS.

## Author Contributions

Y.H. and P.W. performed the research. J.Y. and M.B. designed the research study. L.C. analyzed the data. Y.H. wrote the paper. L.C. and L.W. reviewed and edited the manuscript. All authors have read and approved the final manuscript.

## Ethics Statement

This retrospective study was approved by the Ethics Committee of the Wuxi People's Hospital (KS202056).

## Conflicts of Interest

The authors declare no conflicts of interest.

## Data Availability

The data that support the findings of this study are available from the corresponding author upon reasonable request.
